# Global producer responsibility for plastic pollution

**DOI:** 10.1126/sciadv.adj8275

**Published:** 2024-04-24

**Authors:** Win Cowger, Kathryn A. Willis, Sybil Bullock, Katie Conlon, Jorge Emmanuel, Lisa M. Erdle, Marcus Eriksen, Trisia A. Farrelly, Britta Denise Hardesty, Kristiina Kerge, Natalie Li, Yedan Li, Adam Liebman, Neil Tangri, Martin Thiel, Patricia Villarrubia-Gómez, Tony R. Walker, Mengjiao Wang

**Affiliations:** ^1^ Moore Institute for Plastic Pollution Research, Long Beach, CA 90803, USA.; ^2^ University of California, Riverside, Riverside, CA 92501, USA.; ^3^ Centre for Marine Socioecology, University of Tasmania, Hobart, Tasmania 7000, Australia.; ^4^ CSIRO Environment, Hobart, Tasmania 7000, Australia.; ^5^ Break Free From Plastic, Quezon City 1100, Philippines.; ^6^ School of Urban Studies, Portland State University, Portland, OR 97201, USA.; ^7^ Institute of Environmental and Marine Sciences, Silliman University, Dumaguete City 6200, Philippines.; ^8^ 5 Gyres Institute, Santa Monica, CA 90409, USA.; ^9^ School of People, Environment and Planning, Massey University, Papaioea Palmerston North, Aotearoa, New Zealand.; ^10^ Estonian University of Life Sciences, 51006 Tartu, Estonia.; ^11^ Freelance Researcher.; ^12^ Department of Sociology and Anthropology, DePauw University, Greencastle, IN 46135, USA.; ^13^ Goldman School of Public Policy, University of California, Berkeley, 2607 Hearst Avenue, Berkeley, CA 94720, USA.; ^14^ Global Alliance for Incinerator Alternatives, Berkeley, CA 94704, USA.; ^15^ MarineGEO Program, Smithsonian Environmental Research Center, Edgewater, MD 21037-0028, USA.; ^16^ Facultad Ciencias del Mar, Universidad Católica del Norte, Larrondo 1281, Coquimbo, Chile.; ^17^ Center of Ecology and Sustainable Management of Oceanic Island (ESMOI), Coquimbo, Chile.; ^18^ Stockholm University, Stockholm 114 19, Sweden.; ^19^ School for Resource and Environmental Studies, Dalhousie University, Halifax, NS B3H 4R2, Canada.; ^20^ Greenpeace Research Laboratories, School of Bioscience, University of Exeter, Exeter EX4 4RN, UK.

## Abstract

Brand names can be used to hold plastic companies accountable for their items found polluting the environment. We used data from a 5-year (2018–2022) worldwide (84 countries) program to identify brands found on plastic items in the environment through 1576 audit events. We found that 50% of items were unbranded, calling for mandated producer reporting. The top five brands globally were The Coca-Cola Company (11%), PepsiCo (5%), Nestlé (3%), Danone (3%), and Altria (2%), accounting for 24% of the total branded count, and 56 companies accounted for more than 50%. There was a clear and strong log-log linear relationship production (%) = pollution (%) between companies’ annual production of plastic and their branded plastic pollution, with food and beverage companies being disproportionately large polluters. Phasing out single-use and short-lived plastic products by the largest polluters would greatly reduce global plastic pollution.

## Teaser

Branded plastic pollution is caused primarily by the largest producers of single-use and short-lived plastics.

## INTRODUCTION

Plastic pollution is a globally ubiquitous and increasing problem (*1*–*4*). Plastic products, and associated additives, are harmful to humans (*5*, *6*) and ecosystem health (*7*, *8*). Global plastic production is fundamentally linked to fossil fuel extraction and climate change (*9*–*12*). Plastic production has doubled from approximately 200 million tonnes (Mt) of total production in 2000 to >400 Mt in 2019 (*13*). Increases in plastic production have been accompanied by increases in plastic waste (*13*) and increased volumes of plastics and associated chemicals and byproducts released into the environment throughout the plastic life cycle (*3*, *14*, *15*).

One of the main challenges of addressing plastic pollution is identifying where the plastic products come from (point sources) and who produced them (producers). Point sources have been identified using gradients of increasing waste concentrations (e.g., industrial shipping harbors or roads) (*16*, *17*) or by assessing locations with high concentrations (hotspots) (e.g., plastic-producing factories). There is a growing field of scholarship dedicated to oil spill environmental forensics, involving sampling spilled oil to identify producers of “mystery spills” and including techniques like chemical fingerprinting of crude oil and refined petroleum, petroleum biomarkers, and source apportionment (*18*–*21*). While identifying producers of many chemical pollutants requires sophisticated molecular composition analysis, producers of plastic pollution can often be traced by labels on products (brands) that help to determine their company (the company that owns the brand).

Brand audit events (surveys of brands on plastic waste, also described as audit events or events throughout) are being used to drive producer responsibility initiatives (*22*–*24*). Notably, from 2018 to 2022, brand audit events were conducted across six continents with over 100,000 volunteers following a consistent protocol (*25*). These audit events have suggested that the largest companies in the food and tobacco sectors were the largest polluters in their region. Although this research field is growing, a peer-reviewed strategy for robustly standardizing audit event data does not yet exist.

Economic growth and environmental pollution are inextricably linked in today’s global economic system (*26*), with calls to change economic strategies to avoid pollution (*27*, *28*). Exponential growth in production, consumption, or profit is eventually unsustainable, and degrowth is the solution to overgrowth (*29*). In the past, the discourse on solutions to plastic pollution has largely focused on consumer-based actions and waste management infrastructure instead of producer responsibility (*30*, *31*). However, there is a growing realization that upstream actions by plastic producers have a vital role in addressing the problem (*32*–*34*). Audit event data may indicate problematic products or producer resources to reduce consumer plastic waste and support local material management (*35*–*37*). While data on brands have been used to inform producer responsibility (*23*, *38*), there has yet to be a quantitative analysis of the relationship between plastic production and branded plastic pollution.

Here, we designed a framework for standardizing brand audit event data and linking brands to producers. We then applied the framework to 5 years of the Break Free From Plastic Brand Audit dataset and provide robust quantification of global producers of branded plastic pollution. We hypothesized that plastic production would be linearly related to plastic pollution. We derived the relationship between companies’ plastic production and branded plastic pollution and provided recommendations that could be applied to global policy. These investigations advanced previous research by quantifying global (rather than local or regional) plastic pollution and assessing the relationship between plastic production and plastic pollution generated by individual producers rather than the entire industry. Finally, we made the data and code publicly available to support future analyses and decision-making.

## RESULTS

### Dataset representativeness

#### Spatial

**Fig. 1.**
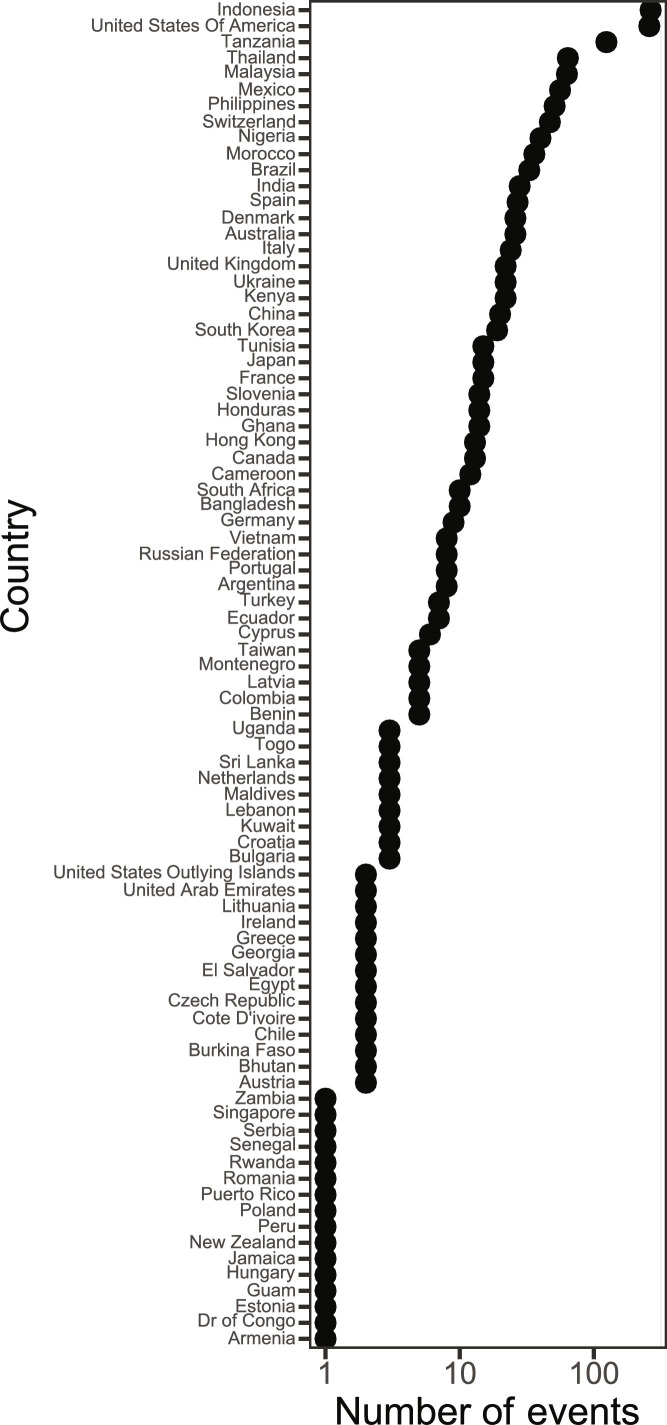
The total number of audit events (*x* axis) that occurred in each country (*y* axis) of the 1576 total. *X* axis is log_10_ scaled to better see the distribution of the event counts per country. There are 84 countries sorted from highest at the top down to lowest. The five countries where the highest number of audit events occurred were Indonesia, United States of America, Tanzania, Thailand, and Malaysia. Dr of Congo, Democratic Republic of Congo.

**Fig. 2.**
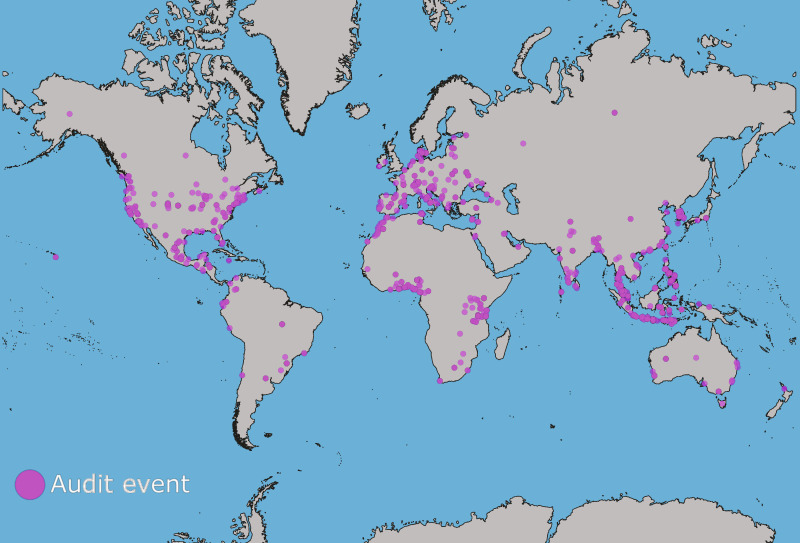
A map depicting where each of the 1576 audit events occurred. Each pink point depicts a single audit event. Points are slightly transparent so that locations with many audits overlapping appear darker. Points are at the most specific location we could ascribe to the audit events. In some cases, that was only at the state or country level, and for those instances, the locations appear as centroids for the country or state. Although there is widespread coverage globally, there are also regions with few data such as South America, central and north Asia, Middle East, and central Africa.

We assessed the spatial coverage of the 1576 (1494 after excluding unbranded items) audit events totaling 1,873,634 (909,771 after excluding unbranded items) documented plastic items to see if they were sufficient for a global analysis (Figs. 1 and 2). Three countries (Indonesia, United States of America, and Tanzania) had over 100 audit events, 29 countries had at least 10 audit events, and 84 countries had at least 1 audit event (Fig. 1) over the 5-year period. The countries used in the analysis represented a combined population of 6.5 billion people or about 81% of the global population, based on July 2022 population estimates (*39*), and accounted for the vast majority of the expected top polluters based on other peer-reviewed literature (*1*, *40*, *41*). Audit events were highly concentrated in Southeast Asia; north, western, and eastern Africa; Europe; and North America (Fig. 2). In this and other studies (*42*), there are noticeable gaps in coverage for South America, central and north Asia, Middle East, and central Africa, which could affect the generalizability of findings, especially for those regions. The data coverage was sufficiently distributed spatially to assess the global mean brand percentages, with previously mentioned caveats.

#### Temporal

Between 237 and 361 audit events were conducted each year. The difference between the minimum and maximum number of audit events conducted is less than a factor of 2 and is unlikely to provide a strong temporal pattern. Hence, data are analyzed together (2018–2022).

#### Unbranded bias

Unbranded plastic items comprised 52% [95% Confidence Intervals (CI_95%_) = 49 to 54%] of the total mean percent. Assigning company ownership to these unbranded plastic items is not possible with current techniques. A similar percent of unbranded plastic has been reported in other studies (*22*). Factors that affect the level of unbranded plastic include inter alia: weathering by water, sun, and air, length of time material is in the environment/frequency of audit events, quality of ink used, and type of material or morphology. Without evidence for the producer identity of unbranded plastic, we focus the following investigation on branded plastic.

### Global producers of branded plastic pollution identified

**Fig. 3.**
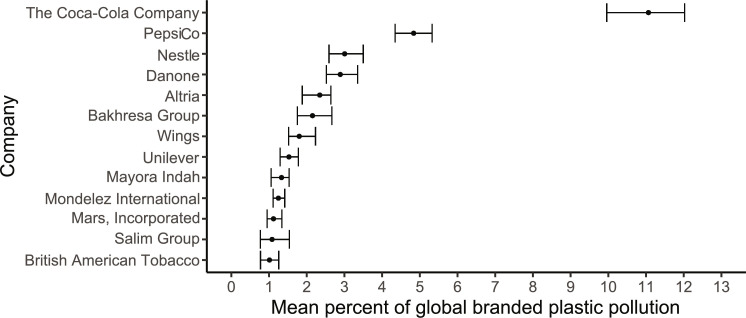
The global percent (*x* axis) of each company’s branded plastic (*y* axis) found in the 1494 audit events. Mean percent is represented as a point, and 95% confidence intervals are represented as whiskers. Where confidence intervals do not overlap, the difference in mean percent is statistically significant. Companies are listed only if their mean percent exceeds 1% of the total branded plastic. Company names can be looked up in wikidata.org. Additional information about company names can be found by searching “[company name] company.” Altria, Altria and Philip Morris International; Wings, PT Wings Surya; Mayora Indah, PT Mayora Indah Tbk.

**Fig. 4.**
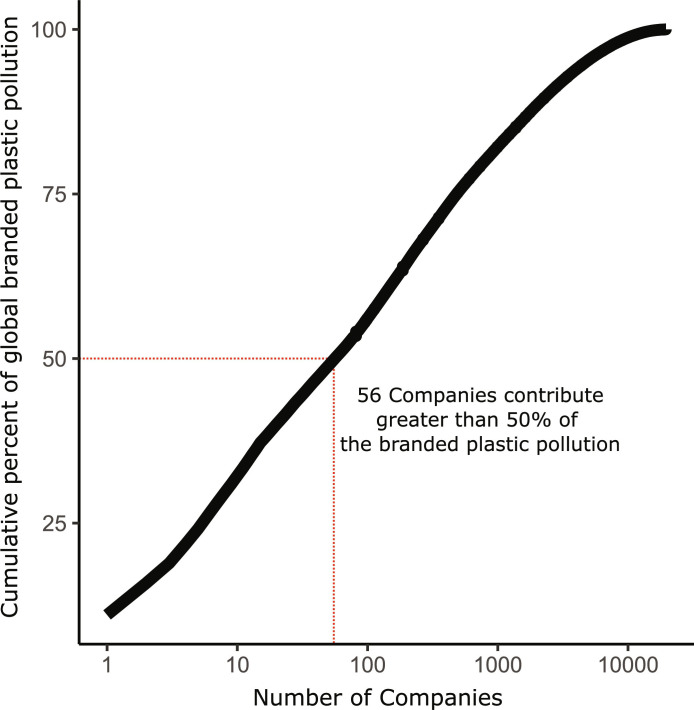
Cumulative percent (%) (*y* axis) of total branded plastic count found during 1494 audit events by the number of companies (count) (*x* axis) responsible for the corresponding cumulative percent. The *x* axis is log_10_ scaled, while the *y* axis is unscaled. The plot shows a log_10_ linear increase in the number of companies involved in pollution with a greater cumulative contribution.

Thirteen companies have an individual contribution of 1% or more of the total branded plastic observed in the audit events (Fig. 3). All 13 companies produce food, beverage, or tobacco products. The top company, The Coca-Cola Company, was responsible for 11% (CI_95%_ = 10 to 12%), significantly greater than any other company. The top 5 companies were responsible for 24% of the branded plastic; 56 companies were responsible for greater than 50% of the branded plastic; and 19,586 companies were responsible for all of the branded plastic (Fig. 4). It is important to note that the contributions of the top companies may be an underestimation because there were brands that were not attributed to a company, and there were many unbranded objects.

### Branded plastic pollution and production relationship

**Fig. 5.**
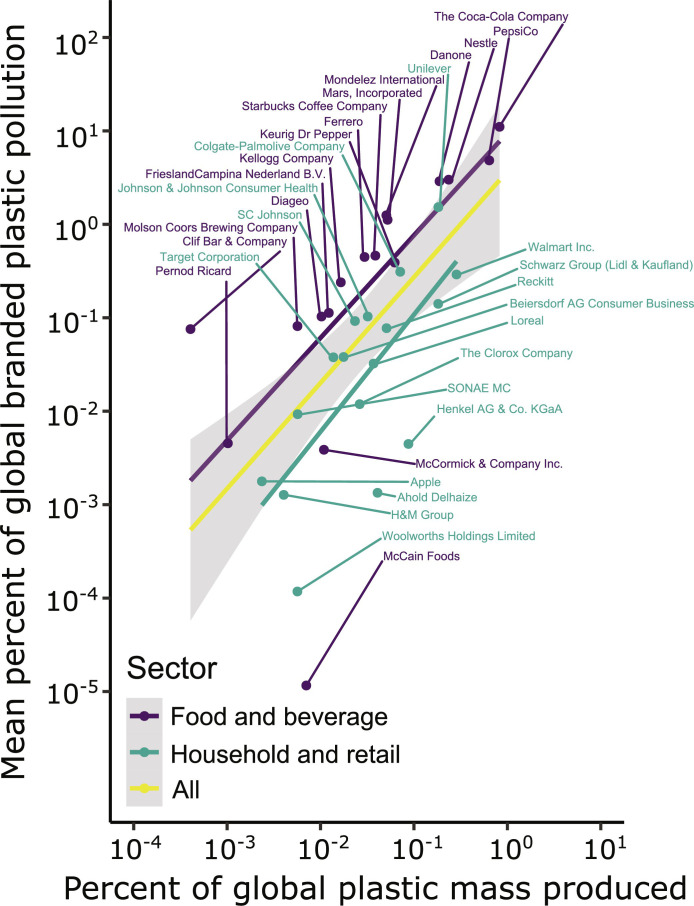
Log-log linear regressions and point plot for the relationship between the percent of global plastic mass produced by companies (*x* axis) and the mean percent of the total branded plastic found in the audit events (*y* axis). Both axes are log_10_ scaled. Points and regressions are colored according to the corresponding industry. Purple is for food and beverage companies, while teal is household and retail companies. The yellow regression line corresponds to the entire dataset, and the gray region denotes 95% confidence intervals. The companies voluntarily reported the total plastic production metric, and the mean percent of total branded plastic was calculated based on Break Free From Plastic Brand Audits. The trend for all data has a slope of 1 (*P* = 5 × 10^−5^), *y* intercept of 0.8 (*P* = 0.4), and adjRSQ of 0.4. The vast majority of companies listed above the trend line are food and beverage companies, and the majority of the companies below are household and retail companies. There was more variability in the relationship for smaller companies, likely due to a lower probability of them in audit events leading to more zero values. Company names can be looked up in wikidata.org to find additional information about them by searching “[company name] company.” SC Johnson, Samuel Curtis Johnson/S. C. Johnson & Son Inc.; H&M Group, Hennes and Mauritz.

To understand the relationship between producer plastic production and pollution, we compared two independently collected datasets: the audit event dataset and the plastic production dataset derived from the Ellen MacArthur Foundation. The log-log linear relationship between both datasets was log_10_(pollution) = log_10_(production) + 0.8, slope *P* value = 5 × 10^−5^, *y*-intercept *P* value = 0.4, adjRSQ = 0.4 (Fig. 5, yellow line “All”), where pollution refers to mean percent of global branded plastic pollution and production refers to percent of global plastic mass produced. The small *y* intercept (0.8) was not significant (*P* > 0.05), which indicates that the *y* intercept is statistically indistinguishable from zero. The slope of 1 can be interpreted as a 1% increase in production, resulting in approximately a 1% increase in branded plastic pollution. This suggests that larger companies are not doing any better or worse than smaller companies at preventing the plastic they produce from entering the environment. The relationship between production and pollution, production (%) = pollution (%), suggests that production is a very strong lever on pollution. Alternative scenarios could exist where larger companies are doing much worse (slope much greater than 1) or larger companies are doing much better (negative slope). In an ideal future scenario, plastic production would be reduced, and this relationship would be a flat line at zero because plastic producers are not polluting.

The companies above the trend line (Fig. 5) were typically food and beverage companies (purple), while the companies below the trend line were mostly household and retail companies (teal). Although both types of companies produce disposable plastic packaging, food and beverage products tend to have shorter time periods of use before disposal, including a higher percent of single-use (inclusive of short-lived) items. Food and beverage products also have a higher likelihood of being consumed on-the-go, while household and retail products have a higher likelihood of being consumed within buildings and are thus less likely to escape materials management infrastructure and leak into the environment. It is important to remember that the percent is count-based. It is possible that this relationship would be different if the percent were for mass because retail and household company products likely have more mass on average than food and beverage company products. Estimates of the mean mass of plastic items produced by each company are required to convert between count and mass.

It is also useful to assess which of the large companies that report to the Ellen MacArthur Foundation are not observed during audit events. Some examples include Amcor (a packaging producer), Jerónimo Martins (a retail grocer), Inditex (a fast fashion company), and Essity AB (a hygiene product company). On the basis of the current information available, we suspect that either their products are much less likely to end up in the environment or their products are not recognizably branded and are partially responsible for the 50% of unbranded plastic recorded in audit events.

## DISCUSSION

The volunteer audit events have value for global plastic pollution monitoring with broad spatial and temporal coverage, which when analyzed can inform policies while enhancing public engagements. A few regions did not have data, and we recommend countries with no audits be prioritized for future data collection. This analysis could be scaled down in future studies to investigate spatially specific mean brand percentages, especially for regions with a statistically robust number of audit events. Future work could investigate how corporate commitments to reduce environmental impact (available in the Ellen MacArthur Foundation dataset) relate to temporal and spatial trends in plastic pollution. Nonplastic items were not investigated in this study but are known to greatly contribute to solid waste, which may become a larger part of the problem if plastics are replaced with alternative materials without addressing the business-as-usual issues causing mass production, consumption, and pollution of materials. We anticipate that if new single-use material production in general does not reduce, alternative materials to plastic could become similarly problematic to plastic today. We recommend that future work be done to assess nonplastic branded items as well. To our knowledge, this is the best global dataset available to science, which can link individual producers to their branded plastic items that end up in the environment. We recommend future studies to build on this benchmark dataset and that it be used as a policy and management tool.

We found over 50% of plastic items were unbranded, highlighting the need for better transparency about production and labeling of plastic products and packaging to enhance traceability and accountability. We suggest creation of an international, open-access database into which companies are obliged to quantitatively track and report their products, packaging, brands, and releases to the environment. Additionally, we recommend development of international standards around the branding of packaging to facilitate their identification.

Core findings of this analysis suggest a paradigm shift in how we regulate plastic producers. The power law relationship (Fig. 4) indicates that a few companies are responsible for half of branded plastic pollution. This suggests that action by these companies, whether voluntary or mandated by governments or an international legally binding instrument, can positively address the problem. The strong linear relationship between plastic production and branded plastic pollution, across geographies and widely varying waste management systems, suggests that reduced plastic production is a primary solution to curb plastic pollution (Fig. 5). Producer brand managers and policymakers should prioritize solutions that reduce plastic production.

The prevalence of food and beverage companies, particularly those that specialize in single-use plastic products, above the trendline in Fig. 5, and retail and household goods companies below the line, suggests that (i) single-use packaging disproportionately contributes to branded plastic pollution and (ii) waste management systems play a role in addressing the problem but are insufficient on their own to eliminate plastic emissions to the environment.

We conclude that effectively addressing global plastic pollution requires corporate producers of plastic waste to reduce plastics in their products and avoid regrettable alternatives, particularly by phasing out nonessential and avoidable single-use products; by safe and sustainable product designs that cut global demand for new products while increasing reusability, repairability, and recyclability; by investing in nonplastic alternatives with proven better safety and environmental profiles; and by supporting alternative distribution models (e.g., refill-reuse), which lessen pollution.

## MATERIALS AND METHODS

### Experimental design

#### Data collection

Each year (5 years total), Break Free From Plastic affiliate surveyors (mostly volunteers) around the globe conducted brand audit events where they surveyed large (>5 mm) solid plastic waste found in the environment (referred to as plastic pollution throughout) in a series of surveyor-selected locations, including beaches, parks, rivers, and other terrestrial systems. Surveyors sorted all plastic pollution from other materials and recorded the brand or trademark on each plastic item and the number of items with those brands wherever possible. The location, date, type of plastic, type of item, number of plastic layers, and time of each audit event were also recorded. The Break Free From Plastic Brand Audit methodology was faithfully applied throughout the study, with the exception of minor changes to the data format and instructions, which did not alter the findings. The data were uploaded to the Break Free From Plastic online repository and validated and aggregated by a database management team.

#### Data cleaning and mapping

Brand data are notoriously challenging to link to companies. Trademarks can be transferred between companies and refer to products owned by several companies in different countries or product sectors. Company names are not always listed on products, and one company can own hundreds of brand names (e.g., PepsiCo owns Lays, 7UP, Quaker, and many more). Globally, brand names can come in any language and can be symbols, making them difficult to read or input into a computer accurately (e.g., Nike’s trademark is a copyrighted swoosh symbol). Companies that own trademarks may be subsidiaries of other companies, and ultimate product ownership is sometimes a holding company (e.g., Berkshire Hathaway). Furthermore, many plastic items are unlabeled or may be so degraded in the environment that labels are unrecognizable, leading to further challenges with identifying producers.

##### Initial brand-company identification

Data collected by Break Free From Plastic Brand Audits link the company to the brand name reported by the surveyors. When surveyors submitted their data, the data submission platform contained a column for inputting the company. The brand-company information was primarily received in this manner from surveyors. The surveyors determined the company either from the packaging directly or looked it up online and entered the appropriate information before finalizing their submission to Break Free From Plastic.

##### Data harmonization with open refine

OpenRefine (*43*) was used to simplify the brand names by converting all letters to lowercase and removing trailing whitespace. Text clustering algorithms were then used to cluster company names that were structurally and phonetically similar (e.g., “Coca-Cola” or “Coca Cola” were clustered to “The Coca-Cola Company”). Last, the Wikidata reconciliation service (www.wikidata.org) was used to automate data enrichment by standardizing company names to their Wikidata identifiers (e.g., PepsiCo = Wikidata ID Q334800).

##### Data prioritization and manual validation

Over 28,570 unique brand labels were represented in the 1576 brand audit events from 84 countries. We created a system for prioritizing a manual labeling effort because we estimated that manually curating the entire dataset would take years of full-time effort. We created a dataset that contained the following variables: brand name, parent company name, countries in which the branded item was recorded, and total item count and total frequency of brand occurrence for each brand name from the total dataset. We used the total item count and total frequency as prioritization values. We set a threshold to ensure we manually validated brands with an item count larger than 100 and a frequency larger than 10 (*n* = 484, which amounted to 66% of the total brand count and 37% of the total brand frequency). Below the threshold, 2668 other brands were manually verified when time and expertise (i.e., from coauthors located in countries where brands had been observed) permitted. During the validation, coauthors assessed whether the brands were linked to the correct companies by researching sources online. This procedure was the most time-intensive of the data-cleaning steps. Company identifiers were issued for each brand based on the following priorities: (i) Wikidata ID, (ii) associated webpage showing that the company owns the brand, (iii) the company name established during Initial Brand-Company Identification, and (iv) brand name reported. If the first identifier priority (i.e., a Wikidata ID) could not be found, then the next identifier in order of priority (i.e., the second identifier, an associated webpage) was used as the company identifier.

##### Final validation

Final data cleaning and standardization were conducted to produce a reusable dataset for statistical analysis in the present study and to support future research efforts. We removed audit events with two or fewer plastic items and user errors, such as inaccurate dates or zero counts from the final dataset. We also removed any event where the recorded percentages for brands from each audit event did not add up to 100%. Last, we removed any audit events of indoor plastic (home audits) to focus on plastic collected in the outdoor audit events, which directly measure environmental plastic pollution. The dataset resulting from the manual verification process (i.e., the dataset containing the verified company identifiers) was joined with the cleaned, full dataset using the brand name as the joining variable.

We tested the cleaned-up dataset with the following automated validation rules to ensure that it conformed to reproducible standards: (i) no blank company IDs, (ii) for each event the proportion of all brands adds up to 100%, (iii) standardized terms for categorical values, (iv) total count for each audit event must be greater than zero, (v) the total item count for each event must be greater than or equal to the total count for any individual brand in this event, (vi) no missing or zero values for brand counts or total event counts or percentages, (vii) rows are distinct, (viii) longitude coordinates are between −180 and 180, (ix) latitude coordinates are between −90 and 90, (x) brand percentages are between 0 and 100, and (xi) every column was type checked to ensure expected data types (e.g., numerical data and categorical data).

### Statistical analysis

R (version 4.3.0) (*44*), Rstudio (*45*), and R packages dplyr (*46*), data.table (*47*), readr (*48*), readxl (*49*), stringr (*50*), ggplot2 (*51*), stringdist (*52*), fuzzyjoin (*53*), wikidataR (*54*), tidygeocoder (*55*), mapview (*56*), sf (*57*), ggrepel (*58*), and ggtext (*59*) were used for statistical analysis and plotting. Inkscape (*60*) was used for fine-tuning of figures. To assess spatial and temporal relationships in the data, we first grouped audit events by country and year, and counted the number of events for each. Audit event locations were then mapped (*56*) in R (*44*) using their most specific spatial domain from the dataset by geocoding the centroids using tidygeocoder (*55*) (city > state > country). City-level specificity was geocoded for 1033 events, state or provincial level for 244 events, and country level for 256 events. Some events (43) could not be geolocated, so while they were not used for spatial analysis, they were included in the global estimates. Events with at least one unbranded plastic recording were used to assess the percentage of unbranded plastic. Events that did not contain at least one unbranded recording were excluded, as some surveyors ignored unbranded plastic, as allowed by the method. Mean unbranded percent was assessed by calculating the percent of unbranded plastic from each audit event, then taking their mean across all audit events, and estimating the uncertainty using bootstrapping (resampling with replacement, *n* = 100, 95% quantiles). Unbranded plastics were excluded from any of the following analyses so that we could calculate the branded percent; this led to the exclusion of 82 audit events, and we used the remaining 1494 for further analyses.

#### Global producers of branded plastic pollution

##### Power analysis

Estimating mean percentages is a well-founded statistical power analysis technique commonly applied to social surveys (*61*). We set the margin of error to 2% because we aimed to statistically differentiate companies that were at least 4% different from one another. We set the expected percent to 10% because previous analyses of this dataset suggested that 10% was the maximum global percent for any company (*25*), and using the maximum percent requires the highest sample size. We set the confidence interval at 95%, a standard threshold in statistical analyses. To avoid pseudoreplication, we considered one sample equivalent to one audit event instead of one branded plastic item. We determined that we needed 857 audit events for the desired statistical power, and 1494 cleaned and verified audit events met this threshold.

##### Bootstrap of mean percentages globally

A technique for estimating the mean percent of waste characteristics was developed to dampen biases inherent in plastic pollution surveys when counts per event were unequal to allow for more accurate and robust statistics (*22*). First, 0% values were added to the dataset for companies that did not occur at an event. Then, a global mean percent was estimated for each company by averaging company percentages across the events. Uncertainties in global mean percentages were estimated by bootstrapping (resampling with replacement 100 times) the aforementioned operation and taking the 95% quantiles of the bootstrap distribution as the confidence intervals. Given the diverse distribution of audit event locations (Fig. 2), a mean was adequate instead of a weighted mean; weighting would have unnecessarily complicated the analysis.

When considering producer responsibility, it is important to describe the scale of the problem in terms of the number of companies involved. We were interested in the minimum number of companies equaling 50% of the total branded plastic found. We sorted the company percentages from greatest to largest and computed a cumulative sum.

#### Branded plastic pollution and production relationship

We aimed to assess the relationship between plastic production and pollution. We used The Global Commitment dataset created by the Ellen MacArthur Foundation (*62*) from data volunteered by companies about their 2021 annual plastic production, in combination with the percent of each company’s plastic pollution described in the Global Producers of Plastic Pollution section. The two datasets had 34 companies in common that we could use for the analysis. Companies not present in both datasets were removed from the analysis and, in some cases, were major polluters (e.g., Altria, Bhakresa Group, Wings, Mayora Indah, and Salim Group). Both datasets were log_10_ normally distributed, satisfying the normality assumption for linear regression. Log-log linear regression was applied to assess the relationship between a company’s global mean pollution contribution as a function of their annual plastic production.
